# *Porphyromonas gingivalis *induces CCR5-dependent transfer of infectious HIV-1 from oral keratinocytes to permissive cells

**DOI:** 10.1186/1742-4690-5-29

**Published:** 2008-03-27

**Authors:** Rodrigo A Giacaman, Anil C Asrani, Kristin H Gebhard, Elizabeth A Dietrich, Anjalee Vacharaksa, Karen F Ross, Mark C Herzberg

**Affiliations:** 1 Department of Diagnostic and Biological Sciences, School of Dentistry, University of Minnesota, Minneapolis, MN 55455, USA; 2 The Mucosal and Vaccine Research Center, Minneapolis VA Medical Center, Minneapolis, MN 55417, USA; 3 Departamento de Rehabilitación Buco-Maxilofacial, University of Talca, Talca, Chile

## Abstract

**Background:**

Systemic infection with HIV occurs infrequently through the oral route. The frequency of occurrence may be increased by concomitant bacterial infection of the oral tissues, since co-infection and inflammation of some cell types increases HIV-1 replication. A putative periodontal pathogen, *Porphyromonas gingivalis *selectively up-regulates expression of the HIV-1 coreceptor CCR5 on oral keratinocytes. We, therefore, hypothesized that *P. gingivalis *modulates the outcome of HIV infection in oral epithelial cells.

**Results:**

Oral and tonsil epithelial cells were pre-incubated with *P. gingivalis*, and inoculated with either an X4- or R5-type HIV-1. Between 6 and 48 hours post-inoculation, *P. gingivalis *selectively increased the infectivity of R5-tropic HIV-1 from oral and tonsil keratinocytes; infectivity of X4-tropic HIV-1 remained unchanged. Oral keratinocytes appeared to harbor infectious HIV-1, with no evidence of productive infection. HIV-1 was harbored at highest levels during the first 6 hours after HIV exposure and decreased to barely detectable levels at 48 hours. HIV did not appear to co-localize with *P. gingivalis*, which increased selective R5-tropic HIV-1 *trans *infection from keratinocytes to permissive cells. When CCR5 was selectively blocked, HIV-1 *trans *infection was reduced.

**Conclusion:**

*P. gingivalis *up-regulation of CCR5 increases *trans *infection of harbored R5-tropic HIV-1 from oral keratinocytes to permissive cells. Oral infections such as periodontitis may, therefore, increase risk for oral infection and dissemination of R5-tropic HIV-1.

## Background

Systemic infection after oral exposure to HIV-1 has been reported in breastfed infants from seropositive mothers [1]. Whether HIV/AIDS is acquired through oral exposure to seminal fluid from HIV-positive individuals remains equivocal [2]. Yet, experimental evidence points to the plausibility that exposure of the oral mucosal epithelium to HIV-1 results in primary infection of the oral tissues followed by systemic dissemination. For example, when simian immunodeficiency virus (SIV) is non-traumatically swabbed on the gingival and buccal mucosa of primates, oral epithelial infection is evident within one day [3,4], while systemic infection occurs within a week [5]. Consistent with these observations, human oral epithelial cells of HIV-infected patients contain integrated HIV-1 DNA, which may result from either primary infection or systemic dissemination of the virus [6]. HIV-1 has also been suggested to infect human oral epithelial cells in vitro [7,8]. Recent work from our laboratory shows that replication aborts after viral integration, while harbored virions are transmissible from oral keratinocytes to permissive cells [9]. In vivo, however, human oral epithelium is generally not considered a target for primary infection by HIV-1 [10,11].

Mucosal exposure is responsible for the vast majority of the current HIV infections worldwide [12] and R5-tropic HIV-1 accounts for most of primary infections [13-15]. In mucosal tissues such as in the gut, CCR5 has been proposed to act as a "gatekeeper", facilitating primary infection by R5-tropic while excluding X4-tropic HIV-1 [14,16,17]. Indeed, primary R5-tropic HIV-1 infection generally requires target cells that carry a specific receptor for gp120 such as CD4 and the chemokine coreceptor CCR5 [18]. Interestingly, a homozygous defect in expression of the R5-tropic coreceptor CCR5 is associated with resistance to HIV-1 infection in frequently exposed individuals [9]. On mucosal surfaces where epithelial cells predominate, the mechanism by which R5-tropic HIV-1 is specifically selected, and X4 HIV-1 is relatively excluded remains unclear. Many potential "gatekeeper" mechanisms have been proposed [17]. More than relying on a single "gatekeeper", selective R5-HIV transmission seems to depend on the aggregate activity of cell and tissue specific restrictive barriers and facilitated uptake mechanisms encountered as HIV-1 passes from the mucosal surface to permissive cells in the organized lymphoid tissues [17].

Healthy squamous oral keratinocytes predominately express CXCR4 [7], but low to undetectable levels of CCR5 [19,20] and there is no expression of the major HIV-1 receptor, CD4 [7,11,21,22]. Given that oral keratinocytes can integrate HIV-1 DNA, alternative HIV-1 receptors have been proposed, including galactosyl ceramide (GalCer) [23,24], heparan sulfate proteoglycans [11,25], syndecans [26,27], and mannose receptor [28,29]. In concert with CXCR4 (X4-tropic HIV-1 specific) or CCR5 (R5-tropic HIV-1 specific) chemokine coreceptors, these alternative receptors have been suggested to take up infectious HIV-1 [30], which can then be transferred to permissive cells [27,30-32].

Since oral epithelial cells express only CXCR4 [7,19,20,22], and oral keratinocytes in vitro can internalize and transfer infectious HIV-1 [22], we sought to learn if CCR5 coreceptor regulation by co-infecting oral bacteria could result in increased uptake and transfer of R5-tropic HIV-1. Co-infecting viruses, such as human herpesvirus 6 (HHV-6) and HHV-7, down-regulate expression of the HIV-1 co-receptor, CXCR4 [33,34]. Since HHV modulation does not affect CCR5, CXCR4 down-regulation may increase the relative expression of CCR5, enhancing the "gatekeeper". Our group has recently shown that *Porphyromonas gingivalis*, an endogenous periodontal pathogen, selectively up-regulates CCR5 in oral keratinocytes [20]. These cells increase CCR5 expression when signaled through protease-activated receptors (PAR) and TLRs by the *P. gingivalis *putative virulence factors, gingipains (Rgp and Kgp) and LPS, respectively [20]. We, therefore, hypothesized that *P. gingivalis *co-infection increases HIV-1 transfer of infectious R5-tropic HIV-1 from oral keratinocytes to permissive cells. In the absence of productive infection in oral keratinocytes, we showed that *P. gingivalis *caused a CCR5-dependent increase in transfer of R5-tropic HIV-1. As a consequence of primary non-productive infection, R5-tropic HIV-1 is suggested to disseminate selectively from oral mucosal epithelium in association with *P. gingivalis *infection in periodontitis.

## Results

### *P. gingivalis*-induced release of infectious R5-specific HIV-1 from oral epithelial cells

**Figure 1:**
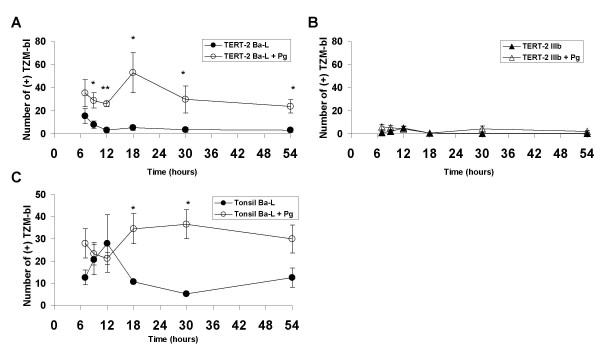
**Assay of infectious HIV-1 virions released by oral epithelial cells**. TERT-2 cells (A and B) and primary tonsil cells (C), with and without *P. gingivalis *pre-incubation, were incubated with (A and C) R5-HIV-1 (Ba-L) or (B) X4-HIV-1 (IIIb) as described in the Materials and Methods. In brief, cell monolayers were incubated with *P. gingivalis *for 3 h, washed, inoculated with HIV-1 and incubated for 3 h, washed and then incubation continued for the total elapsed time as shown. At the indicated times, culture supernatants were harvested from the infected TERT-2 or tonsil epithelial cells and incubated with TZM-bl reporter cells for 2 h. At 2 h, the TZM-bl medium was changed and incubation continued for 24 h. Cells were stained with X-Gal and infected reporter cells per well were counted. Data represent the mean number ± SEM of infected reporter TZM-bl cells per well at the times indicated from 4 independent experiments. * p-value < 0.05, ** p-value < 0.001.

To learn whether *P. gingivalis *might increase release of infectious HIV-1, TERT-2 cells were pre-incubated with *P. gingivalis*, and then inoculated with R5- or X4-tropic HIV-1. Supernatants were collected and presented to reporter TZM-bl cells to assay for infectious HIV-1. From 7 to 54 h post-inoculation, TERT-2 cells pre-incubated with *P. gingivalis *released significantly more infectious R5-tropic HIV-1 into the supernatants than cells incubated with virus alone (Fig. 1A). Release of the X4-tropic strain was unaffected by *P. gingivalis *(Fig. 1B) and was slightly lower than R5-tropic HIV-1, particularly at 7 and 9 h post-inoculation. Like TERT-2 cells, tonsil epithelial cells released significantly more infectious R5-tropic HIV-1 when pre-incubated with *P. gingivalis *(Fig. 1C).

**Figure 2:**
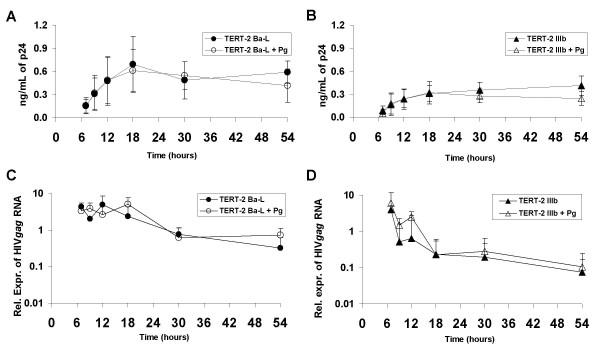
**Total HIV-1 load associated with oral epithelial cells**. (A and B) TERT-2 cells were pre-incubated with and without *P. gingivalis *and then inoculated with R5- (A) and X4-HIV-1 (B). Harvested at the indicated times, supernatants were analyzed for HIV p24 by ELISA. The protocol is as described in the Materials and Methods and summarized in the legend of Fig. 1. Values are the mean of 3 independent experiments and are expressed as ng/mL of p24 ± SEM. (C and D) Expression of HIV*gag *in TERT-2 cells. TERT-2 and TZM-bl cells were pre-incubated with or without *P. gingivalis *and then inoculated with HIV-1 Ba-L (C) or IIIb (D). Total RNA was extracted from the cells, reverse transcribed and used as template in real-time PCR for HIV*gag *RNA. HIV*gag *RNA in TERT-2 cells was expressed relative to the expression in TZM-bl cells at 7 h after HIV inoculation. Beta actin was used as housekeeping gene. Data represent the mean of 3 independent experiments ± SEM.

Since the R5-tropic HIV-1-containing TERT-2 cell supernatants were more infectious when cells were pre-treated with *P. gingivalis*, we sought to learn whether TERT-2 cells released more HIV-1 p24. In the presence or absence of *P. gingivalis*, TERT-2 cells released similar amounts of p24 after inoculation with R5- (Fig. 2A) or X4-HIV-1 (Fig. 2B). From 7 to 18 h post-inoculation, X4- and R5-tropic HIV p24 released from TERT-2 cells increased and then remained constant until 54 h.

### Effect of *P. gingivalis *on TERT-2 cell-associated HIV-1

To determine if *P. gingivalis *increased viral association with TERT-2 cells, RNA from infected TERT-2 cells was recovered between 7 and 54 hours post-inoculation and HIV*gag *RNA was quantified by real-time PCR. In the presence and absence of *P. gingivalis*, greater levels of HIV*gag *RNA were generally recovered from R5- (Fig. 2C) than X4-HIV-1 (Fig. 2D) infected TERT-2 cells.

### *P. gingivalis *effects on HIV-1 replication

**Figure 3:**
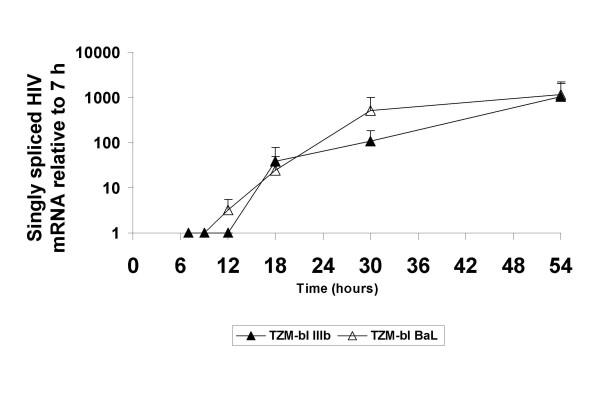
**HIV-1 replication undetectable in oral keratinocytes**. TZM-bl cells and TERT-2 cells were pre-incubated with and without *P. gingivalis *and then inoculated with R5- or X4-HIV-1 strains, washed and incubation continued in fresh medium. The protocol is as described in the Materials and Methods and summarized in the legend of Fig. 1. At the indicated times, total RNA was extracted, reverse transcribed, and analyzed by real-time PCR for the singly spliced gene HIV-1*vpr*. For both cell lines, relative expression of HIV *vpr*-specific singly spliced mRNA (fold-change) is presented in comparison to the levels in TZM-bl cells at 7 h. Beta actin was used as housekeeping gene. Data represent the mean ± SEM of 3 independent experiments. Shown only are data points for TZM-bl cells + HIV-1 IIIb and BaL and TERT-2 cells + HIV-1 BaL. Note that *P. gingivalis *had no effect on the fold-change in HIV *vpr*-specific, singly spliced mRNA.

To determine if *P. gingivalis *affects HIV-1 replication in the oral keratinocytes, TERT-2 cells were pre-incubated with the bacterium and inoculated with either HIV-1 strain. RNA was extracted from the TERT-2 cells and singly spliced HIV-1*vpr *transcripts (newly synthesized mRNA) were quantified by real-time PCR. In the presence or absence of *P. gingivalis*, singly spliced Ba-L and IIIb transcripts were undetectable in the oral keratinocytes for up to 54 h post-inoculation (data not shown). When TZM-bl cells were inoculated directly, however, singly spliced Ba-L and IIIb transcripts increased about 100-fold between 7 and 54 h post-inoculation (Fig. 3). After inoculation with HIV-1 IIIB or Ba-L, TZM-bl cells, but not TERT-2 cells, contained p24gag as shown by immunoblotting (data not shown). These data suggest that there is no replicative cycle of HIV-1 in TERT-2 cells, even when cells are pre-incubated with *P. gingivalis*.

### *P. gingivalis *increases harbored infectious HIV-1 in TERT-2 cells

**Figure 4:**
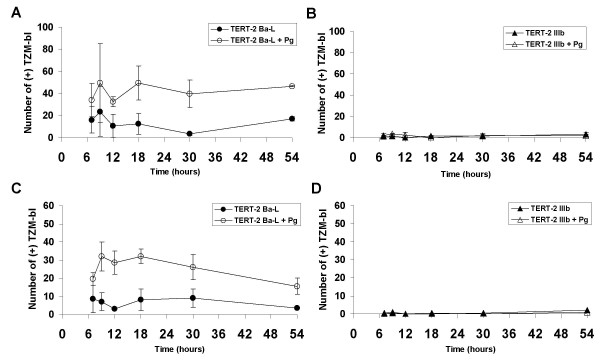
***P. gingivalis *increases infectious HIV-1 associated with oral keratinocyte plasma membrane and intracellular fractions**. TERT-2 cells with or without pre-incubation with *P. gingivalis *were inoculated with R5- (Ba-L) (A and C) or X4-tropic (IIIb) (B and D) HIV-1. The protocol is as described in the Materials and Methods and summarized in the legend of Fig. 1. After washing, cells were trypsinized to recover membrane-associated (A and B) and cell-associated, trypsin-resistant infectious HIV-1 (C and D). To assay for infectious HIV-1 virions, virus-containing fractions were incubated with TZM-bl cells, stained with X-Gal and positive blue cells counted as described in the Materials and Methods. Data represent the mean ± SEM of TZM-bl positive cells from two independent experiments, each in triplicate.

Since *P. gingivalis *pre-incubation caused a selective increase in release of infectious R5-HIV-1 from TERT-2 cells (Fig. 1A), we determined whether infectious virions were internalized or plasma membrane-associated. Oral keratinocytes were pre-incubated with *P. gingivalis *and inoculated with HIV-1 as previously. At times from 7 to 54 h post-inoculation, cultures were washed to remove loosely associated virus and adherent, plasma membrane-associated HIV was detached using trypsin for 5 min. To assess the infectious levels of the detached viral particles, the medium was recovered and the trypsin was inactivated. The infectivity of the recovered HIV-1 was assayed using the TZM-bl reporter cells. Based on the responses of TZM-bl reporter cells, more infectious plasma membrane-associated R5-tropic HIV-1 was detached from TERT-2 cells pre-incubated with *P. gingivalis *than in the absence at all times (Fig. 4A). In the presence and absence of *P. gingivalis*, similar amounts of infectious membrane-associated X4-tropic HIV-1 were detached from TERT-2 cells (Fig. 4B). After removing the plasma membrane-associated virions, cells were lysed to recover internalized HIV-1. Lysates were inoculated onto the reporter TZM-bl cells to assess the levels of infectious intracellular HIV-1 within the oral keratinocytes. Oral epithelial cells pre-incubated with *P. gingivalis *contained more infectious intracellular R5-tropic HIV-1 than *P. gingivalis*-untreated cells (Fig. 4C). X4-HIV-1 inoculated keratinocytes contained barely detectable levels of intracellular infectious virus, which was unaffected by *P. gingivalis *(Fig. 4D). Hence, *P. gingivalis *increases harbored membrane-associated and intracellular R5-tropic HIV-1.

### Increase in TERT-2 cell-associated infectious R5-tropic HIV-1 blocked by anti-CCR5 antibodies

**Figure 5:**
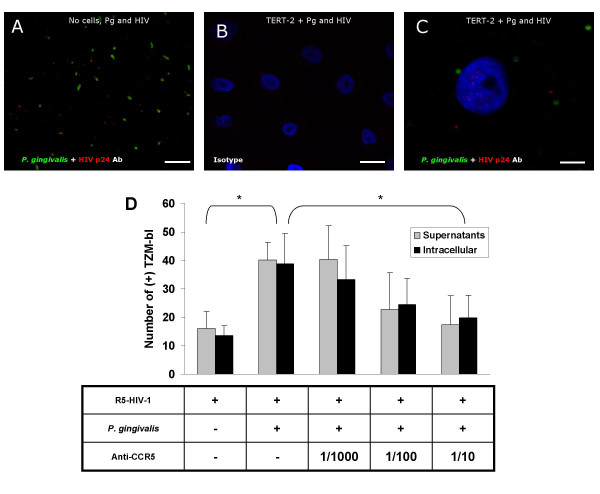
**Increase in TERT-2 cell-associated, infectious HIV-1 independent of direct interactions with *P. gingivalis *and blocked by anti-CCR5**. (A) *P. gingivalis *was co-cultured with R5-HIV-1 on glass slides. (B and C) TERT-2 cells were pre-incubated with *P. gingivalis*, washed and inoculated with R5-HIV (Ba-L), washed, fixed and permeabilized for confocal microscopy analysis. Cells were stained with antibodies (1:100 dilutions) against *P. gingivalis *and HIV p24, or isotype control IgG. The confocal analysis was validated for assessment of intracellular HIV-1 using TZM-bl cells. Color key: Blue, DAPI; Red, Alexa 568; and Green, FITC conjugated IgG. Scale bars: 5 μm (A), 10 μm (B and C). Pictures are representative of 2 independent experiments and show one z-slice through the middle of the nucleus. (D), TERT-2 cells were pre-incubated with *P. gingivalis *or untreated and then incubated with anti-CCR5 antibody for 1 h. All TERT-2 cell cultures were then inoculated with R5-HIV-1. Culture supernatants (gray) and cell lysates (black) were collected at 18 hours post-inoculation with *P. gingivalis *as described in the legend of Fig. 1. Infectious virions were estimated using TZM-bl reporter cells. Data represent the mean ± SEM of 3 independent experiments, each performed in triplicate.

To explain increased cell-associated, infectious R5-tropic HIV-1 fractions (plasma membrane and intracellular), we first considered the possibility that R5-tropic HIV-1 binds *P. gingivalis*, which subsequently invades the oral keratinocytes [35]. TERT-2 cells were pre-incubated with *P. gingivalis*, inoculated with R5-HIV-1 and observed by confocal microscopy. *P. gingivalis *and HIV-1 were also co-incubated on glass slides without cells and then observed. *P. gingivalis *and viruses did not appear to co-localize when co-cultured in the absence (Fig. 5A) or presence (Fig. 5B and 5C) of oral keratinocytes. When pre-incubated with *P. gingivalis*, TERT-2 cells appeared to contain more intracellular HIV-1 (data not shown).

We next tested whether up-regulation of the CCR5 HIV-1 coreceptor on TERT-2 cells [20] by *P. gingivalis *could contribute to the infectivity of R5-tropic HIV-1. Oral keratinocytes were pre-incubated with *P. gingivalis*, then incubated with anti-CCR5 antibody, and inoculated with HIV Ba-L. At 18 h post-inoculation, spent culture supernatants and TERT-2 cell lysates were recovered and assayed for infectivity using TZM-bl reporter cells. Anti-CCR5 caused a dose-dependent reduction in infectious R5-tropic HIV-1 from both the culture supernatants and the intracellular compartment of TERT-2 cells (Fig. 5D). At the highest dose tested, anti-CCR5 blocked the increase in R5-tropic HIV-1 infectivity attributable to *P. gingivalis *(Fig. 5D).

### *P. gingivalis *increases cell-to-cell *trans *infection of intracellular infectious HIV-1 from oral keratinocytes

**Figure 6:**
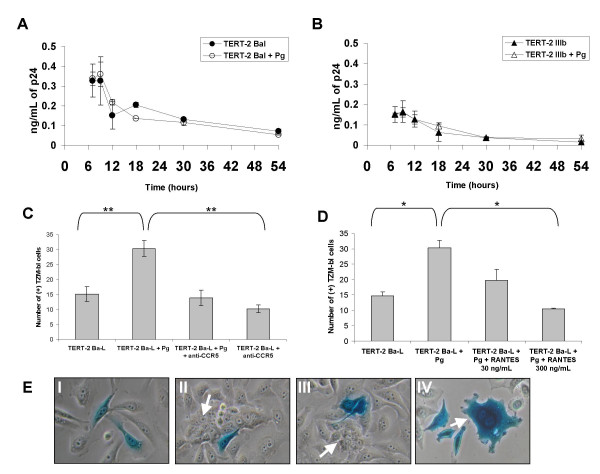
**CCR5-dependent cell-to-cell transfer of infectious HIV-1**. TERT-2 cells were pre-incubated with *P. gingivalis *or untreated and then inoculated with (A) R5- and (B) X4-HIV-1. The protocol is as described in the Materials and Methods and summarized in the legend of Fig. 1. Cells were trypsinized, washed, and lysed. The lysates were assayed for HIV p24 by ELISA. Similarly, TERT-2 cells were pre-incubated with *P. gingivalis *or untreated and then incubated with (C) a 1:10 dilution of 200 μg/mL anti-CCR5 antibody or (D) with 30 ng/mL or 300 ng/mL of the CCR5 inhibitor RANTES, and R5-HIV-1 for 6 h (in the presence of the antibody or RANTES). At 18 h post-inoculation, oral keratinocytes were detached with trypsin, washed twice and seeded onto TZM-bl cells for co-culture. Data represent the mean ± SEM from 3 independent experiments, each performed in triplicate. * p-value < 0.05, ** p-value < 0.001. (E) Photomicrographs of X-gal stained cell co-cultures of TERT-2 cells pre-incubated with *P. gingivalis *and infected with Ba-L for 6 h (I, II and III) as described above. In the co-cultures with TZM-bl cells, arrows identify some proximal TERT-2 cells (panels II and III). Positive control Ba-L-inoculated TZM-bl cells co-cultured with TZM-bl cells are also shown (IV). In panel IV, the arrow shows a multinucleated TZM-bl cell.

Since *P. gingivalis*-pre-incubated cells contained more intracellular infectious R5-HIV-1 than unexposed keratinocytes (Fig. 4C), we studied whether *P. gingivalis *increased HIV entry to the cells. TERT-2 cells were exposed to *P. gingivalis *and both strains of HIV-1 as described previously. Plasma membrane-associated HIV-1 was removed by trypsin and TERT-2 cells were lysed at various times. Consistent with the ELISA data from the culture supernatants (Fig. 2A and 2B), TERT-2 cells pre-incubated in the presence or absence of *P. gingivalis *contained similar intracellular p24^gag ^after inoculation with either R5- (Fig. 6A) or X4-tropic HIV-1 (Fig. 6B). When comparing both viral strains, however, levels of p24^gag ^were higher for R5- than X4-tropic HIV-1 (Fig. 6A, B), which was consistent with the data for cell-associated HIV*gag *RNA (Fig. 2C and 2D) and supernatant p24^gag ^(Fig. 2A and 2B).

We next sought to learn whether oral keratinocytes pre-incubated with *P. gingivalis *transferred more infectious intracellular R5-HIV to permissive cells in co-culture. After trypsinization and removal of extracellular virus, *P. gingivalis *pre-incubated TERT-2 cells were incubated with R5-HIV-1 or X4-HIV-1 for 6 h and cultured for 18 h post-inoculation. At 18 h, infected TERT-2 cells were harvested and co-cultured with TZM-bl reporter cells for an additional 24 h. TERT-2 cells *trans *infected TZM-bl cells with more infectious R5-tropic HIV-1 when pre-incubated in the presence of *P. gingivalis *than in the absence (Fig. 6C). In contrast, X4-HIV-1 *trans *infection from TERT-2 cells to TZM-bl cells was not affected by *P. gingivalis *and was lower than R5-HIV-1 (data not shown).

To determine whether *trans *infection of intracellular R5-HIV-1 was CCR5-dependent, TERT-2 cells pre-incubated with *P. gingivalis *were incubated with anti-CCR5 antibody and then inoculated with R5- or X4-HIV-1. TERT-2 cells incubated with CCR5 antibody and R5- or X4-HIV-1, in the absence of *P. gingivalis *served as negative control. Blocking the TERT-2 cell CCR5 receptor with antibodies significantly reduced *trans *infection of R5-HIV-1 (p < 0.001) (Fig. 6C) but not X4-HIV-1 (not shown) to TZM-bl cells. After pre-incubation with *P. gingivalis*, anti-CCR5 reduced *trans *infection of R5-tropic HIV-1 to levels similar to HIV-1 inoculated TERT-2 cells without *P. gingivalis*.

To confirm the role of CCR5, TERT-2 cells with or without pre-incubation with *P. gingivalis *were incubated with the CCR5 ligand, RANTES, at 30 and 300 ng/mL or with 10 or 100 nM of TAK-779. TAK-779 selectively blocks HIV gp120 interaction with CCR5 [36]. Consistent with the CCR5 antibody data, cells incubated with RANTES (Fig. 6D) or TAK-799 (data not shown) before inoculation with HIV-1 showed statistically significant (p < 0.05) dose-dependent reductions in the increased transfer of R5-HIV-1 mediated by *P. gingivalis*. As expected, TERT-2 cells inoculated with X4 viruses in the presence or absence of *P. gingivalis *were not affected by RANTES or TAK-779 (data not shown). In the absence of HIV-1, TERT-2 cells incubated with either *P. gingivalis*, CCR5 antibody, RANTES or TAK-779, showed no false-positive transfer (staining by TZM-bl reporter) (data not shown).

At harvest, co-cultured TERT-2 cells, which had been washed and trypsinized to remove extracellular and plasma membrane-associated HIV-1, appeared to *trans *infect the TZM-bl reporter cells (Fig. 6E). In some fields, blue TZM-bl cells and TERT-2 cells appeared to grow independently (Fig. 6E, panel I). More commonly, blue TZM-bl cells and TERT-2 cells grew in direct contact (panels II, III). In comparison to co-culture with HIV-1 infected TERT-2 cells, TZM-bl-to-TZM-bl *trans *infection of R5-tropic HIV-1 resulted in 100-fold more infected reporter cells (data not shown), increased multinuclear cells with syncytia formation, and more intense staining (Fig. 6E, panel IV). Although the contact status with TZM-bl cells at the time of *trans *infection was not established, the images suggest that TERT-2 cells *trans *infect either released virus or directly transferred internalized HIV-1 mediated by cell-to-cell contacts. *P. gingivalis*-mediated increased transfer of intracellular HIV-1 was unrelated to reverse transcription. Indeed, AZT (500 μM) maintained continuously in TERT-2 cells cultures did not affect the increase in R5-tropic HIV-1 *trans *infection caused by *P. gingivalis *(data not shown).

## Discussion

Endogenous bacteria may modulate HIV-1 infection. For example, we have shown that the oral endogenous pathogen, *P. gingivalis*, can up-regulate CCR5 on oral keratinocytes [20]. We next sought to learn whether CCR5 up-regulation by *P. gingivalis *could modulate dissemination of R5-tropic HIV-1 from oral keratinocytes. In this report, we show that *P. gingivalis *increases the transmissibility of infectious R5-tropic HIV-1 to proximal permissive cells in vitro without affecting the dissemination of X4-tropic HIV-1. To the best of our knowledge, this is the first report of interactions between septic oral epithelial cells and HIV-1.

HIV-1 infection never occurs in a sterile environment and the septic mucosal environment may affect susceptibility to HIV-1 infection. The oral mucosa and virtually all mucosal epithelial tissues are colonized by polymicrobial biofilms that may modify the acquisition of HIV-1 infection. Co-infecting microorganisms that affect the clinical course of HIV-AIDS or mechanisms of infection include *Mycobacterium tuberculosis *[37], human hepatitis C virus [38], hepatitis B (HBV) [39] herpes simplex virus-2 (HSV-2) [40], *Neisseria gonorrhea *[41], cytomegalovirus, Epstein-Barr virus, HHV-6, -7, and -8, and human papilloma virus [42]. When HIV and *Leishmania *co-infect, the severity increases for both infections [43]. Similarly, the malaria-causing protozoan *Plasmodium *is highly associated with the occurrence [44] and severity of HIV infections [45]. Co-infection with *Mycobacterium avium *may directly increase the severity of infection by increasing HIV-1 replication [46].

*P. gingivalis *is a putative pathogen associated with periodontitis, a polymicrobial infection of the gingiva and tooth-supporting connective tissues, bone and ligament [47]. When challenged with commensal and pathogenic bacteria, oral keratinocytes release cytokines and chemokines [48], which may function as chemoattractants for CD4-positive T cells [49]. Infiltrating CD4+ T cells can then co-localize with keratinocytes, facilitating docking and transfer of infectious virions [49]. In the gingiva, immature dendritic (Langerhans) cells [50] typically co-localize with keratinocytes and T cells [51]. Indeed, we show that TERT-2 cells contact and apparently transfer virus to co-cultured CD4-positive TZM-bl cells (Fig. 6E panels II–III), whereas the virus does not appear to interact directly with *P. gingivalis *(Fig. 5). The data also show that virus released by TERT-2 cells can also be captured by TZM-bl cells (Fig. 6E panel I). TZM-bl cells were used to both assay infectivity of cell-free HIV-1 and serve as a permissive target for *trans *infection of HIV-1 from oral keratinocytes. The results in TZM-bl cells parallel data we have obtained using primary tonsil keratinocytes and several keratinocyte cell lines using peripheral blood mononuclear cells as permissive targets (Vacharaksa et al, unpublished data).

In the presence of *P. gingivalis*, TERT-2 (Fig. 6C, D) and primary tonsil epithelial cells (data not shown) selectively increase *trans *infection of R5-tropic HIV-1 to TZM-bl cells. The selective increase in infectious R5-tropic HIV-1 on the plasma membranes and within TERT-2 cells (Fig. 4), and release into the extracellular environment (Fig. 1A) is independent of new viral replication (Fig. 3). TERT-2 cells appear to take up and contain the same amount of HIV-1 over time in the presence and absence of *P. gingivalis *based on HIV-1*gag *RNA and p24 levels (Fig. 2). The reason for this striking increase in infectivity is not clear. The amount of recovered virus protein or RNA can be discordant with levels of infectious virions [26,52]. Furthermore, non-permissive HIV-1 infection of oral keratinocytes occurs at low frequency, and small differences in the presence and absence of *P. gingivalis *may challenge the sensitivity and discrimination of the detection assays. To estimate viral infectivity, we show that counting 10 to 40 positive cells of the 1 × 10^4 ^TZM-bl cells per well is reproducible and reliable. Infectivity apparently discriminates better than detection or quantification of viral protein. It is clear, however, that the ability of keratinocytes to bind and capture HIV-1, as estimated by HIV*gag *RNA and p24 levels, does not reflect the persistence and transfer of infectious virions to target cells.

Two plausible mechanisms emerge to explain the *P. gingivalis*-mediated selective increase in infectious R5-tropic HIV-1. As a consequence of *P. gingivalis*, TERT-2 cells selectively harbor and protect infectious R5-tropic HIV-1, but not CXCR4-tropic virus. In addition, *trans *infection of R5-tropic HIV-1 to permissive TZM-bl cells also increases in a CCR5 up-regulation-dependent manner. Although both are dependent on pre-incubation with *P. gingivalis*, these mechanisms differ.

In response to *P. gingivalis*, infectious virions were consistently recovered from TERT-2 cell culture medium (Fig. 1A), cell surface (Fig. 4A) and within the cell (Fig. 4C). AZT treatment did not affect viral infectivity suggesting that transfer of intracellular R5-HIV-1 from oral keratinocytes was independent of intracellular viral uncoating or reverse transcription. Harbored HIV-1 remains infectious for up to two days (Figs. 1A, 4A, C). Dendritic cells show similar capability. For example, attachment of HIV-1 to DC-SIGN preserves infectious virus up to 4 days [53]. When compared to cells that do not express DC-SIGN, preservation of viral infectivity results in an increase in *trans *infection to CD4+ permissive cells [53]. The *P. gingivalis*-mediated selective increase in cell-associated R5-tropic HIV-1 suggests a novel protective activity is expressed in oral epithelial cells. This protective activity for harbored HIV-1 may be independent of the expression of CCR5. Protective activity may function directly on R5-tropic virus or by inhibiting HIV-1 inactivation mechanisms. *P. gingivalis *alters the gene expression profile in TERT-2 cells through lipopolysaccharide activation of Toll-like receptors and protease activation of protease-activated receptors [20]. Therefore, modulation of innate immunity by *P. gingivalis *may enable keratinocytes to increase the infectivity of harbored R5-tropic HIV-1.

Perhaps in concert with protection of harbored virus, we also showed that *P. gingivalis*-mediated upregulation of CCR5 in TERT-2 cells [20] increases the effectiveness of *trans *infection to permissive TZM-bl cells (CD4+ CXCR4+ CCR5+). In TERT-2 cells, CCR5 appears to function primarily in *trans*, increasing the delivery of R5-tropic HIV-1 to CD4+ permissive cells. The *trans *function appears to be analogous to DC-SIGN on dendritic cells, which enables *trans *infection to permissive cells [53]. Clearly, CCR5 blockade with specific antibodies, RANTES or a receptor antagonist inhibits the *P. gingivalis*-mediated increase in selective R5-tropic HIV-1 *trans *infection (Figs. 5, 6). The CCR5-dependent increase in *trans *infection of R5-tropic HIV-1 could reflect TERT-2 cell uptake, release or cell-to-cell transfer of virus. That *P. gingivalis *does not affect levels or kinetics of intracellular HIV-1*gag *RNA (Fig. 2C, D) and p24 (Fig. 6A, B) argues against a significant role for CCR5 in selective R5-tropic HIV-1 uptake in these cells. While the data suggest strongly that CCR5 is necessary for the specific *trans *infection of R5-tropic HIV-1, it is likely that internalization of the virus within the keratinocyte is CCR5-independent. Consistent with our findings, blocking CCR5 antibodies reduced transcytosis of R5-specific HIV-1 through primary genital epithelial cells, resulting in attenuated infection of CD4+ cells [27]. Furthermore, up-regulation of CCR5 appears to be necessary, but may not be sufficient for *trans *infection. The net effect of the *P. gingivalis*-mediated up-regulation of CCR5 expression, however, appears to be an increase in the proportion of R5-tropic HIV-1 that successfully transits though the keratinocyte to *trans *infect permissive targets.

Interestingly, *P. gingivalis *proteases, particularly RgpA, inhibit gp120-mediated HIV-1 fusion with the highly permissive MT4 T-cell line and facilitate proteolysis of the CD4 receptor [54]. In the present study, the CD4-negative oral keratinocytes [7,11,21,22] show HIV internalization in the presence of *P. gingivalis *Rgp. Like dendritic cells (DCs) [12], HIV-1 appears to enter CD4- cells by endocytosis, involving clathrin-coated vesicles [55,56] or macropinosomes [57]. Like DCs [53,58], infection of oral keratinocytes is non-productive (Vacharaksa et al, unpublished), but it remains to be learned whether, like DCs [12,59], keratinocytes use synapse formation to *trans *infect. Indeed, recent evidence suggests that HIV-1 enters oral keratinocytes by an endocytic pathway within minutes (Dietrich et al, unpublished) without apparent reliance on gp120 and CD4-mediated membrane fusion [56,57]. If *P. gingivalis *modifies interactions between receptors or co-receptors and HIV-1, candidate targets would be other than cell-fusion associated CD4, CXCR4 and CCR5. Clearly, the interactions between TERT-2 cells and *P. gingivalis *are complex and up-regulation of CCR5 provides only a partial explanation for the increase in trans infection of R5-tropic HIV-1. If these mechanisms simulate pathways in vivo, the oral keratinocyte would be placed in the circuit of transmission of HIV-1, capturing R5-tropic HIV-1 from the mucosal surface and transferring the infectious virus to permissive cells such as infiltrating CD4-positive T cells or specific intraepithelial dendritic cells (DCs). Since iDCs dock with CD4+ T cells, *P. gingivalis*-infected oral keratinocytes can contribute to the selective systemic dissemination of R5-tropic HIV-1.

Collectively, these data suggest that select mucosal sites in the oral cavity such as the periodontal tissues, where organisms like *P. gingivalis *are often abundant in the complex microflora [60], may contribute to the complex set of restrictions and enabling pathways that in aggregate serve as the mucosal gatekeeper system for primary R5-tropic HIV-1 clinical infection [17]. A CCR5-dependent gatekeeper mechanism, or one that is regulated by an endogenous co-pathogen, *P. gingivalis*, has not been previously recognized in oral epithelia. Somewhat analogous to *P. gingivalis *in oral keratinocytes [20], the oral pathogen *Actinobacillus actinomycetemcomitans *increases expression of HIV-1 coreceptors in monocytes [61]. Under conditions of inflammation or infection, polarized human endometrial cells also increase release of infectious HIV-1 to the extracellular compartment [32]. Furthermore, in periodontitis, the epithelial barrier is disrupted [46], increasing the proximity of virus or virus-infected keratinocytes to target T-cells [32,62], release of pro-inflammatory cytokines [63] and activation of TLR-dependent signaling pathways by bacteria [64]. Inflammation in the gingiva and oral mucosa could enhance HIV-1 infection of the oral tissues. For example, certain bacterial pathogens [64] and *E. coli *LPS [65] increase HIV-1 promoter activity by signaling through TLR4. We find no evidence that *P. gingivalis *increase HIV-1 transcriptional activity in TERT-2 cells. In control experiments, we ruled out that *P. gingivalis *and its products in spent bacterial media could activate the LTR promoter in TZM-bl cells (data not shown). Since infectious HIV-1 can be harbored in oral keratinocytes, the squamous mucosal epithelium may also constitute a cryptic reservoir of infection in vivo, which is enhanced specifically for R5-tropic HIV-1 in the presence of *P. gingivalis*. Periodontal disease and other oral infections and inflammatory conditions may, therefore, affect the risk for systemic dissemination of HIV-1 from an oral focus. If this speculation is confirmed, novel therapeutic strategies could be developed to thwart HIV-1 at its point of entry.

## Methods

### Cells

Immortalized human oral keratinocytes OKF6/TERT-2 (TERT-2) [66] were provided by James Rheinwald of Harvard Medical School and cultured essentially as described previously [22]. In brief, cells were grown in 5% CO_2 _at 37°C in keratinocyte serum-free (KSF-M; Gibco), supplemented with 0.3 mM CaCl_2_, 25 μg/mL bovine pituitary extract and 0.2 ng/mL epidermal growth factor (TERT-2 medium). Culture media was changed every two days and cells were subcultured at 60 to 70% confluency (about 5 days). Using an IRB approved protocol, palatine tonsil tissues were obtained from routine tonsillectomies performed at the Hennepin County Medical Center, Minneapolis, MN. Primary tonsil epithelial cells were isolated and cultured using a protocol modified from Oda and Watson [67] as described [67]. In brief, tonsil cells were cultured and the medium was partially replaced (70%) every 2 days. Once the primary culture was established, cells were passaged after 4 days in culture at 70 to 80% confluence. Only cells growing in passage 3 or 4 were used for the experiments. Tonsil epithelial cells were at least 96% epithelial based on flow cytometric analysis using epithelial and fibroblast markers. To serve as a positive control and also as permissive targets for HIV-1 infection, TZM-bl cells (JC53) [68] were obtained from and cultured as recommended by the NIH AIDS Research and Reference Reagent Program, MD.

### Viruses and bacteria

The X4- (IIIb) (AIDS Research and Reference Reagent Program, Division of AIDS, NIAID, NIH: HTLV-III_B_/H9 from Dr. Robert Gallo, Cat. 398)[69] and R5-tropic HIV-1 (Ba-L) (AIDS Research and Reference Reagent Program, Division of AIDS, NIAID, NIH: HIV-1_Ba-L _from Dr. Suzanne Gartner, Dr. Mikulas Popovic and Dr. Robert Gallo, Cat. 510)[70] strains were propagated in peripheral blood mononuclear cells (PBMCs) using the protocols of the NIH AIDS Research and Reference Reagent Program. To estimate the amount of infectious virus, the 50% infection endpoint method (TCID_50_) of Reed-Muench was used [71,72]. TCID_50 _of virus stocks was determined in PHA-activated PBMCs. A multiplicity of HIV-1 infection (MOI) of 0.005 was used to infect the cells (TCID_50 _per cell).

*P. gingivalis*, strain ATCC 33277, was grown under anaerobic conditions in a Coy anaerobic chamber (85% N_2_, 5% CO_2 _and 10% H_2_) at 37°C on Todd-Hewitt agar plates (Difco) supplemented with 5% (v/v) defibrinated sheep blood or in Todd-Hewitt broth supplemented with 5 μg/mL hemin (Sigma) and 1 μg/mL menadione (Sigma). Bacteria were grown in 5 mL of broth for approximately 72 h to an OD_620 nm _of 0.9 to 1.1 (early stationary phase) and counted for determination of the bacterial MOI by the spiral plate method [73].

### Infections with *P. gingivalis *and HIV-1

To serve as targets of HIV-1 infection, TERT-2 or tonsil epithelial cells (1 × 10^5^) were seeded in 24-well plates and grown overnight. Culture medium was removed, replaced with fresh pre-warmed medium and freshly harvested *P. gingivalis *were added at a MOI of 100 for 3 h at 37°C in spent Todd-Hewitt broth to a final volume of 500 μL. After *P. gingivalis *incubation, cells were washed 5 times with Dulbecco's Phosphate-Buffered Saline (DPBS) (Gibco). Negative control cells (unexposed to *P. gingivalis*) were incubated with anaerobic Todd-Hewitt broth, and grown under identical conditions. After removing the bacteria at 3 h, HIV-1 IIIb or Ba-L in 500 μL of medium was inoculated at an MOI 0.005 onto keratinocyte cultures and incubated. At 6 h, cells were washed 5 times with DPBS to remove excess virus, fresh medium was added, and incubation continued for up to 54 h post-inoculation with *P. gingivalis *(48 h after washing to remove HIV-1). At each time point, three different samples were obtained: (i) released HIV-1 in 500 μL of spent cell culture supernatants; (ii) plasma membrane-associated HIV-1, and (iii) intracellular, trypsin-resistant HIV-1. To recover plasma membrane-associated virus (sample ii), cells were treated with 250 μL of 0.05% trypsin-EDTA (Gibco) for 5 min at 37°C. Trypsin was inactivated by addition of equal volumes of DMEM (Mediatech) supplemented with 10% FBS (TZM-bl medium). Cell suspensions were collected, centrifuged at 5,255 × g for 1 min and supernatants were recovered containing membrane-associated HIV that was released by trypsin. The resulting cell pellet containing intracellular HIV-1 (sample iii) was resuspended in 500 μL of TZM-bl medium, lysed by 3 cycles of freezing under liquid N_2 _and thawing at room temperature. Lysis was verified by light microscopy. The three samples were stored at -20°C for infectious virion assay (see below) or p24 ELISA. HIV p24 in cell samples was estimated by ELISA (Beckman-Coulter) as described by the manufacturer.

### TZM-bl reporter assay for infectious HIV

To determine transfer of infectious HIV-1 from oral keratinocytes, TZM-bl cells were seeded at 1 × 10^4 ^cells per well and grown overnight in 96-well plates. The HIV-1 samples (100 μL of a 1:2 dilution) were incubated with TZM-bl reporter cells for 2 h at 37°C in 5% CO_2_, washed 3 times with TZM-bl medium, and incubated for 24 h. TZM-bl cells were then fixed with 100 μL of 0.05% glutaraldehyde for 5 min, and washed 3 times with DPBS. Cells were then covered with 50 μL of X-Gal solution (500 mM potassium ferrocyanide, 500 mM potassium ferricyanide, 0.1 M magnesium chloride and 20 mg/mL X-Gal; all from Sigma) and incubated for 2 h at 37°C in 5% CO_2_. After staining, blue positive cells were counted in each well under a light microscope. To assess specificity of the reporter cell to HIV infection, monolayers of TZM-bl cells were exposed to *P. gingivalis*-exposed and unexposed TERT-2 or TZM-bl supernatants without HIV-1 (negative control). At most, 1 or 2 blue-stained cells per well were detected as a false positive result.

### Co-culture of infected TERT-2 cells with reporter TZM-bl cells

TERT-2 cells were pre-incubated with *P. gingivalis *for 3 h, washed 5 times, incubated with R5-HIV-1 (Ba-L) for 6 h, washed 5 times and incubated in TERT medium for 18 h post-inoculation. Extracellular HIV-1 was then removed by trypsin, cells were washed twice with TZM-bl medium and resuspended in TERT medium. TERT-2 cells (1 × 10^4^) in suspension were added to monolayers of TZM-bl reporter cells per well in a mixture of equal volumes of TERT and TZM-bl media. Co-cultures were maintained for 24 h at 37°C in 5% CO_2 _and stained with X-Gal solution, as above.

### Quantitative PCR

Total RNA was extracted from oral keratinocytes with an AllPrep RNA/DNA Kit (Qiagen) according to the manufacturer's instructions and quantitated using the 2100 Bioanalizer (Agilent Technologies). Total RNA (500 ng) was used as template to make cDNA using the Superscript III First Strand Synthesis System for RT-PCR (Invitrogen). The cDNA was then diluted 1:5 with RNase/DNase free water and 1 μl (5 ng) was used as template in the Platinum SYBR Green qPCR SuperMix-UDG w/ROX (Invitrogen). Quantitative PCR was performed on each sample in triplicate on an ABI7900 HT (Applied Biosystems) and subsequent analysis of the data was obtained using SDS 2.1 software (Applied Biosystems), normalizing all genes to human beta-actin (SuperArray Bioscience), employing the delta-delta CT method of relative quantitation [74]. To characterize the HIV-1 life cycle, singly spliced HIV-1*vpr *transcripts and HIV*gag *RNA primers were used as shown in Table 1 and also [9].

**Table 1:** Primer sequences and PCR conditions

**Target**	**Primer**	**Sequences (5'-3')**	**PCR conditions**
**Gag**	For	CCCATAGTGCAGAACATCCA	50°C, 2 min, 95°C, 2 min, and 95°C, 15 s and 60°C, 30 s, for 50 cycles
	Rev	GGGCTGAAAGCCTTCTCTTC	
**Singly spliced**	M669	GTGTGCCCGTCTGTTGTGTGACTCTGGTAAC	50°C, 2 min, 95°C, 2 min, and 95°C, 15 s and 60°C, 30 s, for 50 cycles
	La 23	GCCTATTCTGCTATGTCGACACC	
β **-actin**	Actin F	ATGGCCACGGCTGCTTCCAGC	95°C, 15 s, 55°C, 30 s, 72°C, 15 s for 30 cycles
	Actin R	CATGGTGGTGCCGCCAGACAG	
**GAPDH**	GAPDH F	GAGTCAACGGATTTGGTCGT	95°C, 15 s, 60°C, 30 s, 72°C, 15 s for 30 cycles
	GAPDH R	TTGATTTTGGAGGGATCTCG	

### Confocal microscopy

TERT-2 cells (2 × 10^5^) were seeded on cover slips in 12-well plates and cultured overnight at 37°C in 5% CO_2_. Cells were incubated for 3 h with *P. gingivalis *ATCC 33277 at MOI 100, washed 5 times with DPBS, inoculated with HIV Ba-L for 2 h at MOI 0.005 and washed 5 more times. TERT-2 cells were prepared for microscopic observation, as described [20]. For some experiments, *P. gingivalis *and HIV Ba-L were co-cultured on glass slides without cells. Cells were incubated with a 1:100 dilution (v/v) of murine anti-HIV p24 IgG1 monoclonal antibody (Chemicon Int., Millipore), murine IgG1 isotype control antibody (BD Biosciences, Pharmingen), or rabbit polyclonal anti-*P. gingivalis *IgG. After antibody incubation, cells were washed 5 times and incubated with ALEXA 568-conjugated donkey anti-murine IgG antibody (Molecular Probes) diluted 1:2000 (v/v) and/or FITC-conjugated goat anti-rabbit IgG antibody (Sigma) (1:1000 (v/v) dilution) and DAPI (Molecular Probes) at a 1:3000 (v/v) dilution for 1 h. Samples were observed using a confocal microscope (Olympus FluoView 1000). From each field, 10 to 30 consecutive sections of 0.5 μm thickness were resolved at 1024 × 1024 pixels, analyzed and pseudo-colored. Co-localization of antigens was determined in single sections from the image stacks. Final images were generated using ImageJ 1.37V software (National Institutes of Health). Microscope settings were kept identical for all images captured.

### CCR5 blocking experiments

CCR5 was blocked on TERT-2 cells using goat anti-human CCR5 polyclonal antibody (CKR-5; Santa Cruz), recombinant human RANTES (CCL5) (Peprotech) or TAK-779 (NIH AIDS Research and Reference Reagent Program, Division of AIDS, NIAID, NIH:TAK-779) [36] essentially as reported previously [20]. In the presence of anti-human CCR5 or RANTES, TERT-2 cells were pre-incubated in the presence or absence of *P. gingivalis*. Cells were then inoculated with HIV-1 Ba-L or IIIb at a MOI 0.005, incubated for 6 h, washed 5 times with DPBS and cultured for 18 h post-inoculation. Culture supernatants or cell lysates were recovered, stored at -20°C and inoculated onto TZM-bl cells to report infectious HIV. In some experiments, TERT-2 cells were trypsinized at 18 h post-inoculation and co-cultured with TZM-bl cells, as described above. Infectious virus from the samples was assayed in TZM-bl cells as described above. Oral keratinocytes in the presence or absence of *P. gingivalis *were used as positive and negative controls, respectively.

### Statistical analyses

Statistical analyses were performed by the Student's *t *test for paired values using GraphPad software. Differences were considered significant at a *p *< 0.05.

## Competing interests

The author(s) declare that they have no competing interests.

## Authors' contributions

RAG conceived the study and designed the experiments, carried out most of the experimental work and drafted the manuscript. ACA performed all the real-time PCR reactions, contributed in the design of the experiments and actively participated in discussions about the study. KHG and EAD helped in the development of the infectivity assays, contributed editorial suggestions to the final versions of the manuscript and participated in helpful discussions. AV developed some of the protocols for the viral infections and the culture of the cells used in this study. KFR helped in the editing of the final versions of the manuscript and contributed to the analysis of the data. MCH advised about the study and its design, critically revised the manuscript, suggested significant modifications to its content and gave final approval to the submitted version.
